# Reply: Emerging evidence of endometrial compaction in predicting ART outcomes

**DOI:** 10.1093/hropen/hoae061

**Published:** 2024-10-11

**Authors:** Hannan Al-Lamee, Gayathri Delanerolle, Dharani K Hapangama, Nicola Tempest

**Affiliations:** Department of Women’s and Children’s Health, Centre for Women’s Health Research, Institute of Life Course and Medical Sciences, University of Liverpool, Liverpool, UK; Hewitt Centre for Reproductive Medicine, Liverpool Women’s NHS Foundation Trust, Liverpool, UK; Liverpool Women’s NHS Foundation Trust, Member of Liverpool Health Partners, Liverpool, UK; Nuffield Department of Primary Care Health Sciences, University of Oxford, Oxford, UK; Department of Women’s and Children’s Health, Centre for Women’s Health Research, Institute of Life Course and Medical Sciences, University of Liverpool, Liverpool, UK; Liverpool Women’s NHS Foundation Trust, Member of Liverpool Health Partners, Liverpool, UK; Department of Women’s and Children’s Health, Centre for Women’s Health Research, Institute of Life Course and Medical Sciences, University of Liverpool, Liverpool, UK; Hewitt Centre for Reproductive Medicine, Liverpool Women’s NHS Foundation Trust, Liverpool, UK; Liverpool Women’s NHS Foundation Trust, Member of Liverpool Health Partners, Liverpool, UK

Sir,

We would like to thank Lin *et al.* (2024) for their interest in our recent *Human Reproduction Open* publication (Al-Lamee *et al.*, 2024) and for raising several important questions which we are happy to have the opportunity to comment on.

In this study, we conducted an evidence synthesis, reporting a consolidation of the existing evidence alongside an interpretation of the results, to answer the question ‘Does endometrial compaction (EC) help predict pregnancy outcomes in those undergoing assisted reproductive technologies?’. This question can be answered using a variety of approaches, and the one that we adopted is an accepted methodology. Our approach was an evidence synthesis rather than a narrower approach to answer a single question; hence, we used the well-established PRISMA (Preferred Reporting Items for Systematic Reviews and Meta-Analysis) (Page *et al.*, 2021) and PICO (Population, Intervention, Comparison, Outcome) methods (Higgins *et al*., 2024), both of which are outlined within our systematic review protocol that was prospectively registered in PROSPERO (Al-Lamee *et al.*, 2024). Whilst we recognize the limitations and confounders posed by the available evidence, which were discussed in detail within our article (Al-Lamee *et al.*, 2024), the investigation of factors contributing to infertility, as suggested by Lin *et al.* (2024) was not aligned with our research question. Owing to the wide variations in the inclusion/exclusion criteria between the studies, it was not possible to stratify these further and draw meaningful conclusions. Heterogeneity, due to differences between reported pregnancy outcomes, definition of EC, method of ultrasound and cycle protocol may have accounted for the lack of translation between the clinical pregnancy rate (CPR)/ongoing pregnancy rate (OPR) and live birth rate (LBR) findings; thus, all pooled data should be viewed within this context rather than as a stand-alone point. To explore more granular details, a prospective study would be required and, as described within our study protocol, this was not our aim. The factors highlighted by Lin *et al.* (2024) should certainly be considered when designing and conducting a robust randomized controlled trial in the future. However, in reality, the group of study participants included in our article (Al-Lamee *et al.*, 2024) do reflect common diverse patient cohorts seen within real-world fertility clinics and, therefore, is applicable to clinical practice.

With regard to publication bias, this is an accepted issue within the scope of scientific research. To address the impact of publication bias within the scope of the analysis conducted, we recognize the importance of publication bias being both identified and reported on transparently. Whilst this can be performed with Egger’s test, as suggested by Lin *et al.* (2024), in our study, we opted to use a validated and well-regarded risk of bias tool, the Newcastle–Ottawa Scale (Wells *et al.*, 2000). Additionally, a sensitivity analysis can either be performed on a single or multiple outputs; therefore, the standard approach used within our article was aligned with the good practice guidelines for an evidence synthesis (Marušić *et al.*, 2020).

As stated by Lin *et al.* (2024), we do acknowledge that our systematic review and meta-analysis includes both prospective and retrospective cohort studies, and this has been discussed as a limitation within our article (Al-Lamee *et al.*, 2024). We included 7 prospective and 14 retrospective cohort studies; therefore, the suggestion from Lin *et al.* (2024) to perform sub-group analyses or meta-regressions based on different study designs if there are at least 10 studies in an analysis does not apply. It is important to remember that systematic reviews and meta-analyses often serve as key tools for synthesizing evidence across studies to guide clinical practice, public health interventions, or policy decisions. They can draw from different study types, including clinical trials and epidemiological studies, each offering distinct advantages and limitations (McKenzie *et al.*, 2024). Whilst many systematic reviews focus solely on clinical trial data, this approach presents a limitation by excluding valuable epidemiological data.

Finally, we thank Lin *et al.* (2024) for sharing their thoughts regarding other published meta-analyses looking at the association between EC and pregnancy outcomes (Chen *et al.*, 2023; Turkgeldi *et al.*, 2023; Feng *et al.*, 2024). When our manuscript was initially submitted to *Human Reproduction Open* for review, no meta-analyses had yet been published on the topic. Since then, other systematic reviews and meta-analyses have indeed been published; however, all have included fewer studies and therefore analysed less data than ours, which may have accounted for the differences in our overall findings on CPR and OPR (Al-Lamee *et al.*, 2024). Nevertheless, our conclusions on LBR and miscarriage rate are in keeping with other meta-analyses on the topic. Each of the three other published meta-analyses (Chen *et al.*, 2023; Turkgeldi *et al.*, 2023; Feng *et al.*, 2024) looking at EC and pregnancy outcomes have used different methods, research questions, and outcomes and have included different studies. We appreciate that there are many other ways to conduct a systematic review and meta-analysis; however, each methodology used has its own merit (and, in turn, disadvantages).

In summary, we have used an accepted objective method to answer our specific stated research question that, at the time of writing, includes more data than any of the other published meta-analyses.
